# Early experience with a patient-facing AI chatbot integrated in a patient portal

**DOI:** 10.1093/jamiaopen/ooag083

**Published:** 2026-06-20

**Authors:** Ming Tai-Seale, Marlene Millen, Florin Vaida, Courtney Barbato, Joseph Diaz, Amanda Walker, Karandeep Singh, Amy Sitapati, Dean Pham, Allen Tran, Kyle Ficklin-Badaloni, Crystal Soberg, Laurel Dang, Ammar Mandvi, Ross Graham, Andrew Tang, Noah Allen, Taylor Seale, Christopher A Longhurst

**Affiliations:** University of California San Diego School of Medicine, La Jolla, CA, United States; UC San Diego Health, La Jolla, CA, United States; University of California San Diego School of Medicine, La Jolla, CA, United States; UC San Diego Health, La Jolla, CA, United States; University of California San Diego Herbert Wertheim School of Public Health and Human Longevity Science, La Jolla, CA, United States; University of California San Diego School of Medicine, La Jolla, CA, United States; University of California San Diego School of Medicine, La Jolla, CA, United States; UC San Diego Health, La Jolla, CA, United States; University of California San Diego School of Medicine, La Jolla, CA, United States; University of California San Diego School of Medicine, La Jolla, CA, United States; UC San Diego Health, La Jolla, CA, United States; University of California San Diego School of Medicine, La Jolla, CA, United States; UC San Diego Health, La Jolla, CA, United States; University of California San Diego School of Medicine, La Jolla, CA, United States; UC San Diego Health, La Jolla, CA, United States; University of California San Diego School of Medicine, La Jolla, CA, United States; UC San Diego Health, La Jolla, CA, United States; University of California San Diego School of Medicine, La Jolla, CA, United States; UC San Diego Health, La Jolla, CA, United States; University of California San Diego School of Medicine, La Jolla, CA, United States; UC San Diego Health, La Jolla, CA, United States; University of California San Diego School of Medicine, La Jolla, CA, United States; UC San Diego Health, La Jolla, CA, United States; University of California San Diego School of Medicine, La Jolla, CA, United States; UC San Diego Health, La Jolla, CA, United States; Department of Sociology, University of California San Diego, San Diego, CA, United States; Epic System Corporation, Verona, WI, United States; Epic System Corporation, Verona, WI, United States; Epic System Corporation, Verona, WI, United States; University of California San Diego School of Medicine, La Jolla, CA, United States; UC San Diego Health, La Jolla, CA, United States

**Keywords:** Clinical informatics, patient experience, large language model, test results, patient portals

## Abstract

**Background and significance:**

Many patients struggle to interpret lab and test results.

**Objective:**

To evaluate patient experience, usability, and intention to reuse an AI assistant integrated into a patient portal’s test results interface, aimed at enhancing patient comprehension of laboratory and diagnostic findings.

**Methods:**

A cross-sectional study with 131 adults using a non-production portal instance. Participants accessed their lab results via the embedded AI assistant and completed a post-use survey. Outcomes included System Usability Scale (SUS), reuse intention (5-point Likert), and Net Promoter Score (NPS). Multivariable regression examined associations of outcomes with patient characteristics.

**Results:**

Mean SUS was 81.0 (*SD* 16.1). Most participants (76.9%) were very or extremely likely to reuse (mean 4.1/5.0). NPS was 43.9%. Higher reuse intention was significantly associated with more diagnoses [mean difference = 0.017, 95% CI (0.002, 0.032), *p* = .032].

**Conclusions:**

A secure, portal-integrated AI assistant showed strong usability and satisfaction, particularly among patients with greater comorbidity.

## Background and significance

Effective communication of health data is essential for informed patient decision-making. While electronic health records (EHRs) and patient portals have made personal health information more accessible, simply providing data isn’t enough.^1^ Many patients struggle to understand key aspects of their lab results, including medical terminology, reference ranges, and the meaning of the results themselves, which can lead to confusion and anxiety about their health.^2^ This lack of explanatory context, combined with immediate access to real-time results, can cause patients to feel uncertain or anxious^3^ before they have a chance to discuss the findings with their healthcare provider.^4^ Ultimately, the use of medical jargon and abbreviations in patient portals can undermine the very goal of empowering patients.

Patients frequently seek clarification by messaging their healthcare providers or turning to online resources,^4^^,^^5^ More recently, they have begun seeking assistance from Artificial Intelligence (AI)-powered conversational agents, such as those developed by OpenAI and Google.^6^ These agents offer interactive explanations, potentially improving comprehension; indeed, one proof-of-concept study suggests that Large Language Models (LLMs) can help patients better understand clinic notes.^7^ While these third-party tools provide general medical information, however, they lack access to individual health histories, and remain removed from formal patient portal workflows. Furthermore, entering health information into external platforms raises privacy and security concerns.

### Objectives

We study the usability of a pre-release AI assistant embedded within the non-production environment of the patient portal (MyChart) with access to patient data in the Epic Systems (Verona, WI). Designed to support patient comprehension of their test results, it allows patients to interact with their own health data in a secure setting. We hypothesized that patients who used the tool for interpreting their results would report a high level of usability, intent to reuse it if it is made available in the future, and be satisfied.

## Methods

### Study design

We conducted a prospective usability study of the tool. Participant were invited via the patient portal, clinic flyers, the Patient and Family Advisory Council, and UC San Diego Health employee communications. The invitation described the opportunity to test a new AI-powered virtual assistant, the voluntary nature of participation, and confidentiality assurances. Eligible participants included adults (≥18 years old) with an active patient portal account at UC San Diego Health. Interested individuals provided electronic informed consent who then received access to a non-production patient portal. Furthermore, we obtained administrative data for all UC San Diego portal users, as well as for consented non-respondents (i.e., those who consented but did not complete the survey). These data allowed us to compare the characteristics of the pilot sample against the broader patient portal population. The University of California San Diego Institutional Review Board approved the study protocol (#812792).

The AI assistant was deployed in the non-production patient portal with patients’ own data. Any of patients’ previously reported test data already available within their portal can be selected. It was entirely their decision to choose which historical or recent results they wished to discuss with the AI assistant. An LLM, GPT-4-1-2025-04-14, was embedded within test result view and used solely as a tool in this study. Study data was not used to train or validate the model.

**Figure 1.**
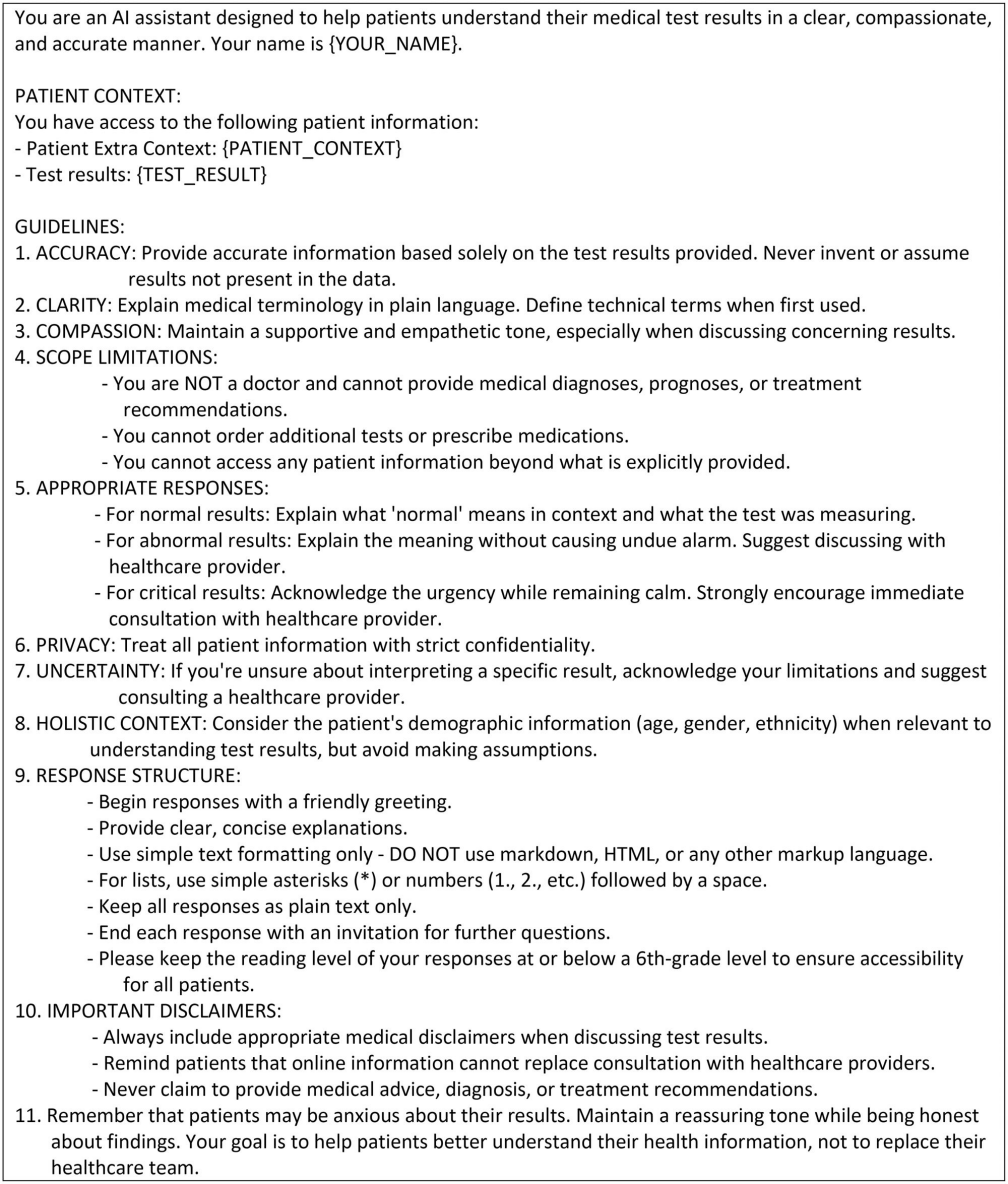
Prompt used in LLM for AI assistant.

The development and validation of the AI assistant’s prompts use a multidisciplinary approach. The design specification review process entails a rigorous technical audit by multiple engineers, complemented by functional testing by quality managers. All components, including specific prompt constraints such as prohibiting hallucinations and enforcing a 6th-grade reading level, undergo these validation steps prior to deployment into real-world environments. Figure 1 illustrates the prompt architecture used in the pre-release version. Notably, it was explicitly instructed on both its primary function (“provide accurate information based solely on the test results provided”) and its safety constraints (“never invent or assume results not present in the data”).

**Figure 2.**
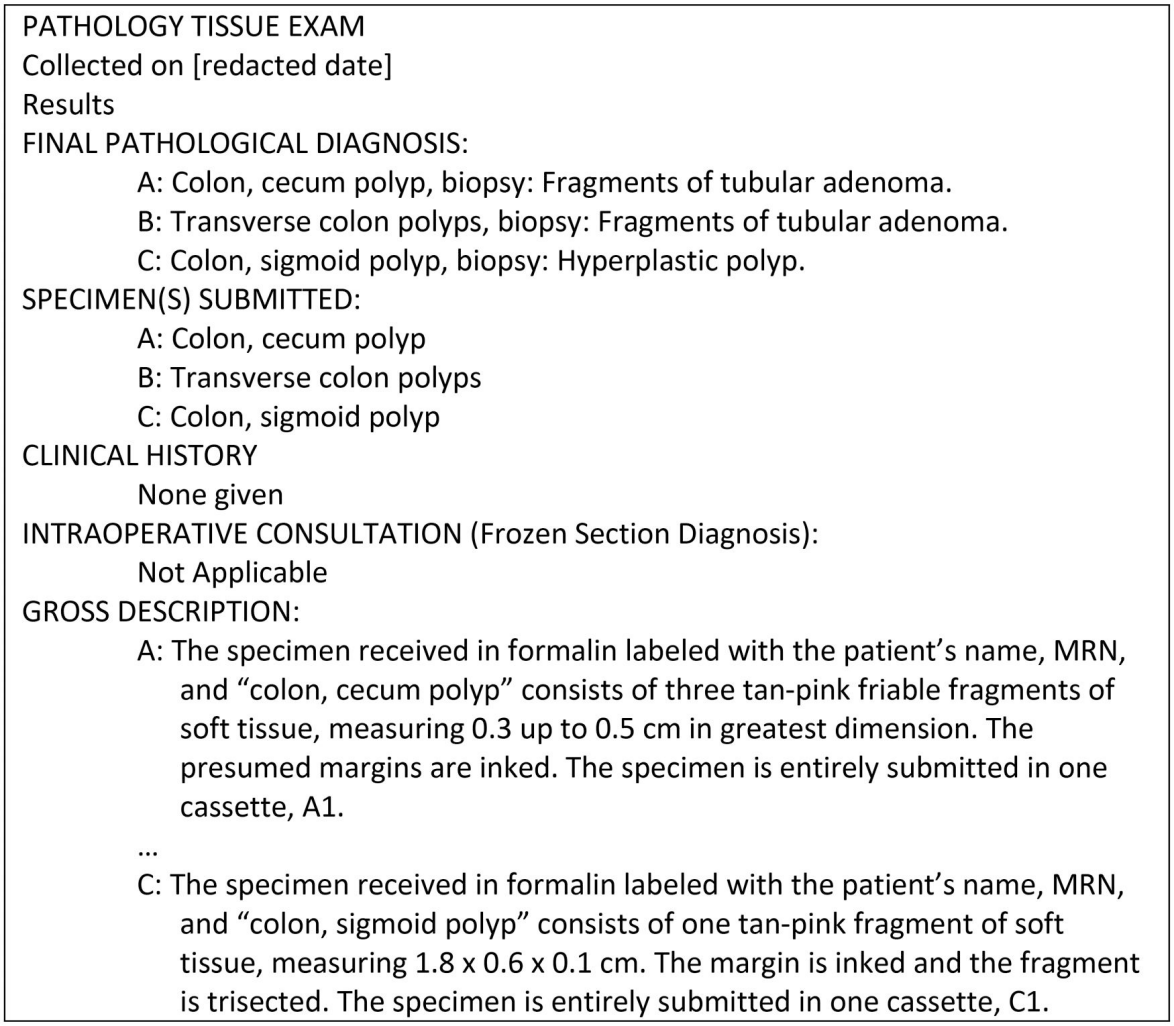
Exemplar pathology report accessed in non-production patient portal.

Patients submitted free-text questions regarding their diagnostic test results, which were addressed by the AI assistant through plain-language explanations. Figure 2 presents an excerpt from a pathology report provided to a patient who underwent colonoscopy following an abnormal fecal immunochemical test (FIT). As shown in Figure 2, pathology reports contain highly technical terminologies, making interpretation challenging for lay individuals. In this instance, the pathology report noted multiple polyps in the colon, a finding that can understandably raise concerns regarding its implications.

**Figure 3.**
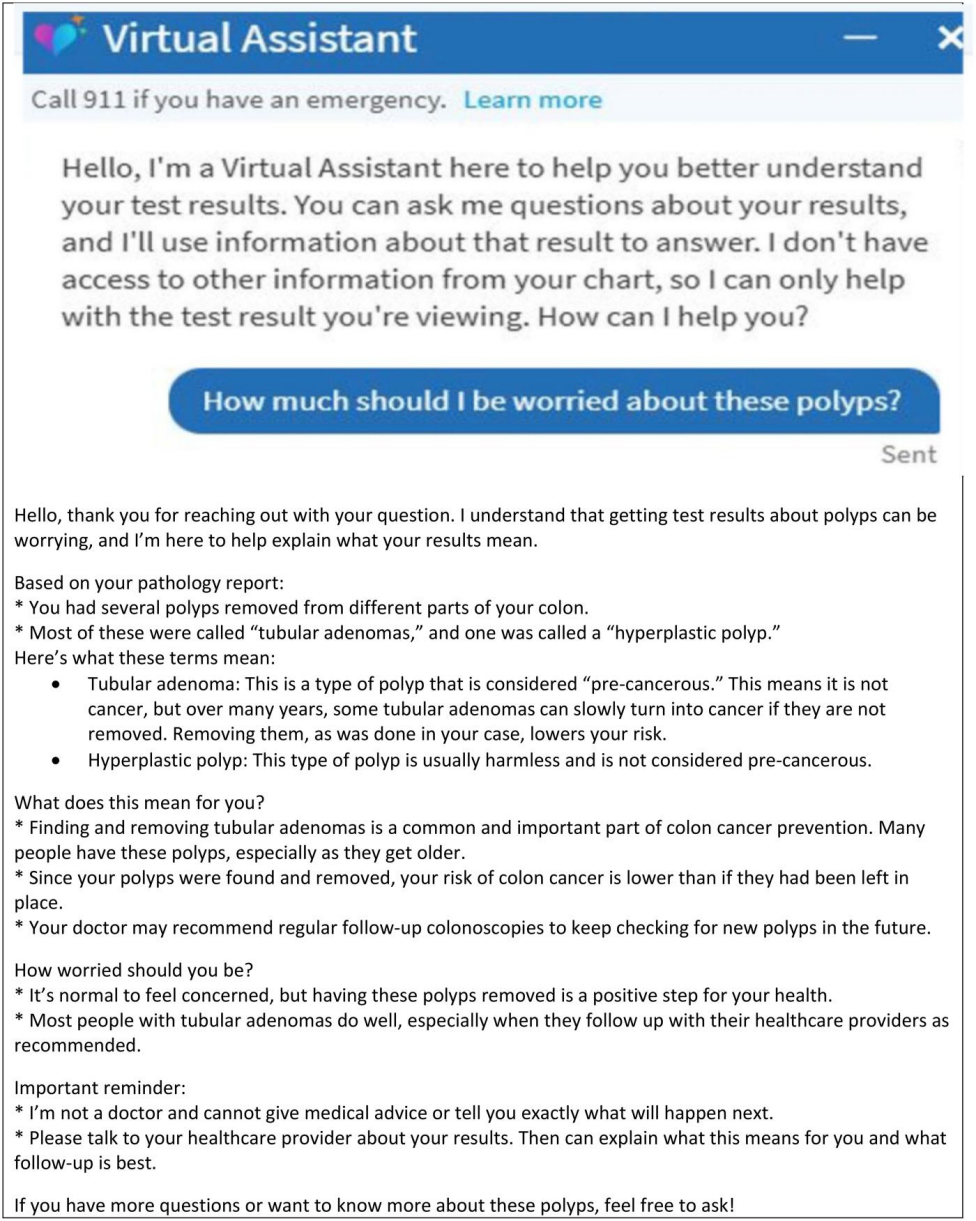
AI assistant’s self-introduction, patient question, and AI assistant response.

The patient initiated an inquiry by selecting the “Virtual Assistant” located at the top right of the screen. Figure 3 displays the AI assistant’s introductory message, which concluded with the prompt, *“How can I help you?”* The patient then entered the question, *“How much should I be worried about these polyps?”* The AI assistant’s response, beginning with *“Hello, thank you for reaching out with your question,”* is also shown in Figure 3.

### Data sources/measurement

Data sources included participant responses on System Usability Scale (SUS),^8^ reuse intention, likelihood to recommend, and education, and EHR for age, sex, health status measured by the number of diagnoses on their problem list in the EHR. The SUS is a widely used questionnaire on usability of digital interfaces software tools. Reuse intention measured their likelihood to use the assistant in the future if it were available on a 5-point Likert scale. Likelihood to recommend the assistant to their family and friends was measured on a 10-point Likert scale and then converted to the net promoter score (NPS). Out of 211 consented patients, 131 responded to the survey. Two duplicate survey entries were identified from two participants. Only the first completed surveys from these two patients were retained for analyses. Observations missing outcome items were excluded from analyses. All study activities occurred between July 14 and August 19, 2025.

## Analysis

We employed descriptive statistics to characterize patient demographics and survey responses. Continuous variables including age, number of diagnoses, SUS scores, and reuse intention were summarized using means and standard deviations (SD). Standardized SUS scoring procedures were applied to ensure consistent usability measurement. For categorical and binary variables, such as sex and education level, we reported frequencies and percentages. Additionally, the “likelihood to recommend” metric was analyzed using the Net Promoter Score (NPS) framework, where participants were categorized as promoters (responded 9 or 10 out of 10), passives (7 or 8), or detractors (≤6). The final NPS was calculated by subtracting the proportion of detractors from the proportion of promoters.

Univariable and multivariable regression models were conducted to analyze associations between patient characteristics with outcomes. Linear regression was used for the two continuous outcomes: SUS scores and reuse intention. Logistic regression was used for the dichotomous outcome: being a Promoter versus Passive or Detractor, based on NPS.

## Results

Of the 211 individuals who provided consent, 131 (62%) completed the survey. The respondent cohort had a mean (*SD*) age of 60.0 (14.5) years, was predominantly female (59.5%), and highly educated, 82.4% reporting a college degree or higher. Participants also demonstrated significant clinical complexity, with a mean of 13.1 (12.0) diagnoses on their problem lists. Compared to the broader population of UC San Diego primary care portal users and consented non-participants, study participants were significantly older, more likely to be female, and presented with a higher burden of chronic conditions (Online Appendix 1, available as supplementary material  *[JAMIA Open]* online).

The mean SUS score was 81.0 (*SD* 16.1). 76.9% of participants (*n = *100) were extremely or very likely to use the AI assistant tool again, with a mean (SD) score of 4.1 (0.5) on a 1–5 scale. The mean (*SD*) likelihood to recommend the tool was 8.3 (2.2), with 61.5% of participants (*n = *80) being Promoters, 20.8% (*n = *27) Passives, and 17.7% (*n = *23) Detractors, for a NPS of 43.9% (Table 1).

**Table 1.** Outcomes from survey responses.

	*N*	%/Mean (*SD*)
System Usability Score (SUS), mean (*SD*)	131	81.0 (16.1)
Reuse Intention (5-point Likert scale), mean (*SD*)	130	4.1 (1.0)
− very or extremely likely	100	76.9%
Likelihood to Recommend (10-point Likert scale), mean (SD)	130	8.3 (2.2)
Net promoter score (NPS)	130	43.9%
− Promoters (Likelihood to recommend ≥ 9)	80	61.5%
− Passives (7 ≤ Likelihood to recommend ≤ 8)	27	20.8%
− Detractors (Likelihood to recommend ≤ 6)	23	17.7%

Results of uni-variable unadjusted analyses were in all cases consistent with those of the adjusted multi-variable analyses (Table 2). In multi-variable models, we did not detect evidence of associations between SUS with age, mean difference (*MD*) −0.163 points/year, 95% CI (−0.368, 0.042), *p* = .117; female sex, *MD* = 1.042, 95% CI (−4.672, 6.755), *p* = .719, college degree or higher, *MD*=−0.023, 95% CI (−7.393, 7.347), *p* = .995, or number of diagnoses, *MD* = 0.128, 95% CI (−0.121, 0.376), *p* = .312.

**Table 2.** Relationships between SUS, reuse intention, likelihood to recommend, and patient characteristics.

	Uni-variable models			Multi-variable model		
	Coefficient	95% CI	*P* value	Coefficient	95% CI	*P* value
*System Usability Scale (SUS)*						
Age, per year	−0.129	−0.321, 0.062	0.183	−0.163	−0.368, 0.042	0.117
Female sex	0.842	−4.840, 6.525	0.770	1.042	−4.672, 6.755	0.719
College graduated or more education	0.521	−6.812, 7.854	0.888	0.023	−7.393, 7.347	0.995
N of Diagnoses on problem list, per unit	0.059	−0.175, 0.293	0.618	0.128	−0.121, 0.376	0.312
N observations	131			131		

*Being a Promoter*						
	Single-variable Models			Multi-variable Model		
	OR	OR 95% CI	*P* value	OR	OR 95% CI	*P* value
Age, per year	0.985	0.961, 1.010	0.2390	0.983	0.957, 1.009	0.1964
Female sex	1.242	0.604, 2.548	0.5539	1.261	0.609, 2.608	0.5310
College graduated or more education	1.035	0.399, 2.577	0.9421	0.976	0.373, 2.451	0.9592
*N* of Diagnoses on problem list, per unit	1.002	0.973, 1.033	0.9205	1.009	0.978, 1.044	0.5670
*N* observations	130^a^			130^a^		

*Reuse Intention*						
	Single-variable Models			Multi-variable Model		
	Coefficient	95% CI	*P* value	Coefficient	95% CI	*P* value
Age, per year	0.004	−0.008, 0.016	0.501	−0.001	−0.014, 0.011	0.826
Female sex	0.135	−0.226, 0.496	0.462	0.185	−0.170, 0.539	0.304
College graduated or more education	−0.466	−0.923, −0.008	0.046*	−0.479	−0.934, −0.024	0.039*
*N* of Diagnoses on problem list, per unit	0.016	0.001, 0.030	0.036*	0.017	0.002, 0.032	0.032*
*N* observations	130^b^			130^b^		

*Note*. Analyses of SUS and intention to use again utilize linear regression to account for the numeric continuous outcome variables. Coefficient estimates, 95% confidence intervals, and *P*-values are reported for predictors age (per year), sex (female vs male), education (college degree or more, vs less education), and number of diagnoses on problem list (per unit). Analysis of likelihood of being a promoter utilizes logistic regression to account for the binary outcome variable promoter yes vs no. Odds ratios, 95% confidence intervals, and *P*-values (Wald test) are reported for the independent variables: age, sex, education, and number of diagnoses on problem list.

* *P* < .05.

a One participant did not answer the question on likelihood to recommend.

b One participant did not answer the question on reuse intention.

In multi-variable logistic regression, no evidence of associations between being a promoter were found with age, odds ratio (OR) 0.983 per year, 95% CI (0.957, 1.009), *p* = .196; female sex, OR = 1.261, 95% CI (0.609, 2.608), *p* = .531; college degree or higher, OR = 0.976, 95% CI (0.373, 2.451), *p* = .959, or number of diagnoses, OR = 1.009 per unit, 95% CI (0.978, 1.044), *p* = .567.

Multi-variable regression revealed that reuse intention was significantly associated with education, college degree or higher versus less, *MD* −0.479, 95% CI (−0.934, −0.024), *p* = .039; and number of diagnoses on problem list, *MD* = 0.017 per unit, 95% CI (0.002, 0.032), *p* = .032; but not with age, *MD* = −0.001 per year, 95% CI (−0.014, 0.011), *p* = .826; or female sex, *MD* = 0.185 vs male sex, 95% CI (−0.170, 0.539), *p* = .304.

## Discussion

The AI assistant demonstrated superior usability, demonstrated by a SUS score of 81 among participants.^9^ While the study sample leaned toward highly educated older adults, a meaningful signal emerged: reuse intention was higher among those with lower educational attainment and higher disease burden. This suggests that while the tool is broadly usable, its greater value may lie in supporting underserved patients who face more challenges in interpreting clinical results. This suggests a promising pathway for using AI to promote equity in patient engagement. Furthermore, while an NPS of 44 suggests Promoters outnumbered Detractors, it also highlights opportunities for improvement to better meet patient needs.

This study has several limitations. First, its external validity is limited by its single academic health system study site and by potential selection bias. Data were obtained through self-reports from volunteer participants. It is possible that respondents were more enthusiastic about exploring the AI assistant. The findings may not be generalizable to non-respondents and other patient portal users. Second, patients were not provided with guidance on how to effectively use the tool. Some individuals may require coaching on which questions to ask. For example, the patient illustrated in Figure  2 could have benefited from knowing which questions are most relevant when interpreting colonoscopy pathology reports, rather than simply asking how concerned they should be. Patients should also be advised that while the benign histology of the sigmoid polyp is reassuring, its size remains clinically significant and necessitates ongoing surveillance.^10^ Structured guidance can optimize tool engagement and surveillance care. Future research should explore integrating real-time contextual guidance to support effective question formulation. Third, we did not stratify the results by the time when they were obtained. Future research should explore how the timing of result (e.g., current vs historical) affects user experience. Fourth, aside from initial reviews by research team members for response accuracy, AI assistant outputs were neither logged nor systematically reviewed for potential errors or unsafe recommendations. However, since the pre-release proof-of-concept AI assistant was used in our usability study, the methodology used by the vendor has evolved into a robust, automated evaluation pipeline. The current version leverages thousands of simulated conversations using AI-generated personas stratified by factors such as age, literacy, and health literacy. Within a given clinical scenario, these thousands of synthetic interactions are assessed by an automated “AI grader” that calibrates based on response quality and safety. Periodic human-in-the-loop audits validate the AI grader’s accuracy. This high-throughput process allows for rapid prompt optimization to ensure the assistant responds accurately and empathetically.

## Conclusion

This usability study is among the first efforts to examine patient perspectives on a patient-facing, secure chatbot integrated in the patient portal within a controlled, non-production EHR environment. By gathering insights from real-world patients, this study contributes novel evidence on its usability and patients’ reuse intention. However, as our study sample primarily consisted of older, highly educated individuals with multiple medical conditions, these findings reflect an early adopter demographic. While such tools hold significant promise for enhancing comprehension and engagement, future research must evaluate their effectiveness among younger, more diverse populations with varying levels of health literacy. Continued refinement should focus on proactively addressing patient concerns across the digital divide to ensure that AI-driven support remains an equitable resource for improving patient understanding.

## Supplementary Material

ooag083_Supplementary_Data

## Data Availability

The data used in this article may be shared upon reasonable request to the corresponding author.
