# Supplementary material for: Early experience with a patient-facing AI chatbot integrated in a patient portal
Source: JAMIA Open. 2026 Jun 20;9(3):ooag083. doi: 10.1093/jamiaopen/ooag083 (PMC13282707; doi:10.1093/jamiaopen/ooag083)
Supplement: ooag083_Supplementary_Data [file ooag083_supplementary_data.docx]

**Online Appendix Table 1. Respondents and Non-Respondents Characteristics**

|  | Consented Patients | | | | All MyChart Users | |
| --- | --- | --- | --- | --- | --- | --- |
|  | Respondents | | Non-Respondents | |  |  |
|  | N | %/Mean (SD) | N | %/Mean (SD) | N | %/Mean (SD) |
| **Patient characteristics** | 131 |  | 80 |  | 602,496 |  |
| Age in years, mean (SD) | 131 | 60.0 (14.5) | 80 | 61.2 (14.7) | 602,496 | 48.7 (21.3) |
| Sex | 131 |  |  |  |  |  |
| - Female | 78 | 59.5% | 46 | 57.5% | 263,355 | 43.7% |
| Education |  |  |  |  |  |  |
| - High school or less | 4 | 3.1% | NA | NA | NA | NA |
| - Some college | 19 | 14.5% | NA | NA | NA | NA |
| - College graduated or more | 108 | 82.4% | NA | NA | NA | NA |
| N of diagnoses on problem list, mean (SD) | 131 | 13.1 (12.0) | 80 | 12.5 (8.9) | 602,496 | 5.0 (8.4) |

Note: NA = Not available
